# Developing Responsive Indicators of Indigenous Community Health

**DOI:** 10.3390/ijerph13090899

**Published:** 2016-09-09

**Authors:** Jamie Donatuto, Larry Campbell, Robin Gregory

**Affiliations:** 1 Swinomish Indian Tribal Community, 11404 Moorage Way, La Conner, WA 98257, USA; lcampbell@swinomish.nsn.us; 2 Institute for Resources, Environment and Sustainability, The University of British Columbia, Vancouver Campus, Vancouver, BC V6T 1Z4, Canada; robin.gregory@ires.ubc.ca

**Keywords:** Indigenous health, health assessment, indicators, evaluation, cultural competency, environmental health justice

## Abstract

How health is defined and assessed is a priority concern for Indigenous peoples due to considerable health risks faced from environmental impacts to homelands, and because what is “at risk” is often determined without their input or approval. Many health assessments by government agencies, industry, and researchers from outside the communities fail to include Indigenous definitions of health and omit basic methodological guidance on how to evaluate Indigenous health, thus compromising the quality and consistency of results. Native Coast Salish communities (Washington State, USA) developed and pilot-tested a set of Indigenous Health Indicators (IHI) that reflect non-physiological aspects of health (community connection, natural resources security, cultural use, education, self-determination, resilience) on a community scale, using constructed measures that allow for concerns and priorities to be clearly articulated without releasing proprietary knowledge. Based on initial results from pilot-tests of the IHI with the Swinomish Indian Tribal Community (Washington State, USA), we argue that incorporation of IHIs into health assessments will provide a more comprehensive understanding of Indigenous health concerns, and assist Indigenous peoples to control their own health evaluations.

## 1. Introduction

This paper introduces a community health evaluation methodology using a unique set of Indigenous Health Indicators (IHI), which focus on a range of health-based considerations that are often overlooked by health assessments conducted in Indigenous communities. In general, health indicators are measures or characteristics of the status of human health in a community. Examples of commonly used indicators are rates of heart disease, infant mortality, and cancer rates. Researchers most often gather health data in order to make generalized statements about the health of a population, and to use the findings in guiding regulatory actions, policy development and health intervention decision-making [1,2]. Health assessments consist of a curated grouping of health indicators, chosen based on the context of the assessment. In the healthcare industry, the focus of health assessments depends on the healthcare practitioner’s field (e.g., physical therapy, oncology, mental health) [3]. Many other types of health assessments exist, some pertinent to the work discussed here; these include: human health risk assessments (HHRA) such as those used to determine increased morbidity and mortality outcomes due to individual exposures to toxicants [4]; community health assessments (CHA) borne from the public health field, which employ definitions of health beyond the physiological, such as housing and education [5]; and, health impact assessments (HIA) that seek to evaluate health on a community-wide scale and to include social and cultural indicators [6]. While some of these assessment types are specific to promulgated regulations in the United States of America, others are part of analogous policies world-wide. 

Many scholars have demonstrated that how health is defined and evaluated is essential to the efficacy of the health assessment findings and related outcomes [7], and in particular for Indigenous peoples [8,9,10] (“Indigenous peoples and nations are those which, having a historical continuity with pre-invasion and pre-colonial societies that developed on their territories, consider themselves distinct…and are determined to preserve, develop and transmit to future generations their ancestral territories, and their ethnic identity, as the basis of their continued existence as peoples, in accordance with their own cultural patterns, social institutions and legal system” [11]). At minimum, a defensible and inclusive health assessment needs to reflect an understanding of the key values and priorities of the people in question and a mechanism for communicating these values to external decision makers (e.g., regulators, industry, elected officials). In order to operationalize such a health assessment framework, the assessment must be driven and refined by the people in question for use in their own communities, reflecting their own unique histories and place-based uses of resources [7,10,12]. 

Health within many Indigenous communities is characterized by a combination of practices and knowledge about coexistence with other human beings, with nature and local natural resources, and with spiritual beings. Current health assessment frameworks lack the ability to recognize and equitably incorporate these Indigenous health values [13,14]. Health assessments may be too narrow in scope (e.g., only focus on physiological health, leaving out cultural, emotional and environmental health and interconnections), limited in scale (e.g., only focus on individuals rather than family or community), or are unable to find and utilize indicators that depict the “intangible” aspects of Indigenous health (e.g., importance of ceremonies, relations with animate objects such as mountains or water [15]). The failures of health assessments for Indigenous peoples have been documented for more than 30 years [8,9,10,13,14,16]. Yet to this day, there are no widely accepted Indigenous models for the conduct of health assessments [17,18]. 

The purpose of this paper is the development of improved measures that may be helpful when evaluating Indigenous community health, keeping in mind that each Indigenous community is unique and even neighboring communities may have differing health values and priorities. The objectives of this paper are to present: (a) the status of Indigenous community health assessments; (b) a methodology for identifying more detailed, context-specific Indigenous health risks and impacts; (c) an innovative set of Indigenous Health Indicators (IHIs) that evaluate Indigenous priorities and are understandable to both Indigenous and non-Indigenous ways of thinking; and (d) results from pilot-tests of the IHI with the Swinomish Indian Tribal Community (Washington State, USA). 

### 1.1. Status of Health Assessments with Indigenous Communities

There are numerous general strategies for incorporating Indigenous health concerns into health assessments. Yet there is no consensus as to what is meant by their “incorporation”. For some, the idea of bringing measures of Indigenous health into evaluations is viewed as a necessary step, so that the concerns of Indigenous communities will be heard by non-Indigenous decision makers such as governments and industry. An Evaluation Report published by the U.S. Environmental Protection Agency’s Office of the Inspector General, for example, provides an overview of suggested procedures for improving the effectiveness of human health risk assessments for clean-up actions taken on tribal lands [19], yet none of the suggestions adequately embody foundational cultural aspects such as the connection between people and their land. Health Canada’s HIA guidelines highlight the importance of Indigenous knowledge and values in health assessments, but do not provide methodological guidance for equitably incorporating Indigenous knowledge and values into a HIA [20]. Without clear, detailed methodologies of how to equitably incorporate Indigenous health values and concerns into a non-Indigenous health assessment framework, the Indigenous values run the risk of co-optation, or becoming invisible within a distinctly unfriendly and unfamiliar health assessment format, where the emphasis remains on physical health impacts to individuals. Not only do these health assessment polices omit key facets of Indigenous health, but the indicators that are used often disregard key cultural values, beliefs and practices [17,21]. 

Critiques of the health assessment frameworks currently applied in Indigenous communities come from a wide range of disciplines. Risk analysts have criticized the failure to include Indigenous-specific exposures levels when calculating health risks, e.g., [22]. To date, most efforts to improve health assessment frameworks focus on the physiological exposure piece of the risk equation. Examples of explicated exposure pathways unique to Indigenous peoples include the harvest and use of culturally important resources such as salmon, wild rice, reeds for basket weaving, or the use of water for sweat lodges, e.g., [23,24]. 

Social science scholars have criticized existing frameworks for failing to address important social and psychological health factors and for omitting many locally experienced social consequences of harm, e.g., [17,25,26]. Some of the specific critiques point out that health within Indigenous communities is often defined on a familial and community scale, rather than individual, and is inextricably connected to the land, waters, air, natural resources, and the maintenance and transfer of Indigenous knowledge [8,9,10,12]. There are recent examples of progress to address these concerns. New Zealand’s Whanau Ora Health Impact Assessment modified a HIA to reflect beliefs, values, and practices specific to the Maori [27]. Other researchers created a map-based approach for evaluating Maori human-environment resource use relationships [28]; however, this approach lacks health measures that are commensurate to broader community health assessments. In Canada, the Regional Health Survey for First Nations, Inuit and Metis communities uses an HIA approach based on an Indigenous cultural framework [29] to assess a wide range of concerns including housing, economic health, infant mortality, smoking, access to healthcare, and language [30]. These approaches, all based on HIAs, work well for some specific uses, but collectively demonstrate the dearth of consistent Indigenous health measures and methodological guidance, which has led to concerns about the quality and consistency of results [31]. 

### 1.2. From Theory to Application

Much has been published on the definition and elicitation of Indigenous knowledge, including similarities and differences between Indigenous knowledge and western-based science, and the pros and cons of combining information from different knowledge systems [11,32,33]. We emphasize that caution must be given to taking Indigenous knowledge out of the context in which it is based, or trying to fit piecemeal bits of Indigenous knowledge into other knowledge frameworks—neither effort proves fruitful [32,33]. Only through fostering a strong, communicative relationship, based on the principles of “meaningful consultation” and “free, prior and informed consent” (Intellectual Property Rights laws and standards for the protection of Indigenous knowledge are set at the international and national (U.S.) level [34,35]), in which all players come to the table and have equal parts in the decision-making process, will any sort of collaboration between Indigenous and non-Indigenous knowledge holders be operational [36,37]. 

The Indigenous Health Indicators (IHI) process is founded on Indigenous knowledge, and combines Indigenous and non-Indigenous knowledge methods in data collection and analysis. The outcome of the assessment is for use in policy and regulations created and primarily managed by non-Indigenous peoples. Thus, the relationships between knowledge sources and uses are complex, and must be acknowledged at the onset in order for the process to achieve its goals. It must also be noted that the authors designed the IHI to be employed in parallel with other health assessments already in use, not for the IHI methods or results to be incorporated into an established health framework, which may not provide means for IHI results to be equitably considered alongside predominant, conventional health indicators. Meeting the practical challenges of a more inclusive assessment framework, including pervasive constraints on financial and technical resources, requires that the set of indicators is sufficiently broad enough to encompass unique Indigenous health values, adaptable in order to be used by many different Indigenous communities, and yet concise to be achievable and useful to decision-makers. A listing of hundreds of concerns may be descriptively accurate but it quickly turns into a reconnaissance mission casting so wide a net that it becomes unwieldy to use as part of a decision-making process characterized by multiple stakeholders and tough trade-offs [38]. Yet the indicators also need to be detailed enough to reflect important health benefits and risks; if not, then important considerations will be missing and participants will not recognize their values in the results [15]. 

The practical constraints associated with using indicators of Indigenous health as part of evaluations and analyses ensure that the indicators (and their measurements) will be incomplete. Indicators are designed as tools to aid in providing a more comprehensive community health assessment, yet they can never fully express the depth or magnitude of the “intangible” values and connections between the human, environmental and spiritual worlds [18]. For some researchers and communities, the shortcomings of an incomplete assessment provide evidence of a lack of concern or, worse, continuation of a “colonial mentality” (Indigenous people as inferior and not deserving of attention or resources). Others have argued that the improvements in health assessments, however partial, are still useful in the decision-making process because they provide information to decision makers about some of the “intangibles”, which would otherwise be omitted, or relegated to an aside as “anecdotal” information. The role of indicators here is to gain awareness and understanding of aspects that had remained outside the purview of assessments, and less about providing exacting, quantitative measures [39]. 

The authors posit that as long as those involved understand that the goal of the IHI is to improve evaluation results (and that no data are released without free, prior and informed consent), and that results will never be fully comprehensive, then evaluations using IHI can aid communities in more effective health planning and decision-making. Decision-aiding frameworks are practical in nature and require such simplification, with experience showing that improvements come from the ongoing examination of their use over time and the incorporation of both methodological and process refinements [40]. 

### 1.3. Sources of Information and Data Quality

As with any careful evaluation, multiple sources of information are generally needed to complete an IHI assessment. A range of tribal government staff, Indian Health Service staff, academics, and consultants may be called upon to provide information for the assessment. The fundamental input, however, comes from local members of the Tribe or Nation, who empower the process by defining what health means to them and by tailoring the IHI to reflect their community’s values and beliefs. Other information can come from field studies, models, or from previous work that has been done in other similar contexts. In all cases, the quality of the data should be checked and, to the extent possible, similar questions should be asked (e.g., in terms of the number of observations, over what length of time they’ve been made) regardless of its source [36].

No matter how carefully information is collected, however, it will be subject to various sources of uncertainty. For example, in the context of a health impact assessment for a proposed resource development initiative, such as a new oil pipeline or the cleanup of contaminants that were buried improperly, uncertainty will be associated with the outcomes of potential actions—how many fish are expected to die in the event that a spill occurs near a river, or what levels of a contaminant will remain in a river after the agency’s cleanup is completed? Uncertainty also will be associated with the probabilities that identified beneficial or adverse events will take place, such as the number of new jobs that are available within a community due to a resource development project, or the time it will take to get equipment to the site after an accidental spill takes place [41]. Other sources of uncertainty are based in external events, not directly related to the proposed project and its direct impacts, which nevertheless might be important with respect to the overall or cumulative effects on community health; a common example is uncertainty with respect to the effects of climate change on the future availability or health of a valued species [42]. 

Although not the focus of this paper, there are at least three main ways in which uncertainty relating to health assessments in Indigenous communities can and should be improved. First, information about the uncertainty associated with the anticipated consequences of actions should be acknowledged whenever the related health impacts may prove to be significant. Second, the range and best estimate of anticipated outcomes should be stated explicitly [43]. Third, as the uncertainty associated with the outcome of actions increases, it becomes more important for decision-makers to consider robust or adaptive management strategies that reflect, rather than obscure, the existing level of knowledge [44]. 

### 1.4. Study Context

The IHI embody over a decade of work initiated by the Swinomish Indian Tribal Community (Swinomish). Swinomish is a U.S. federally recognized American Indian Tribe, signatory to the 1855 Treaty of Point Elliott. The Tribe occupies the Swinomish Indian Reservation on the southern portion of Fidalgo Island in Washington State, which contains approximately 7000 upland and 3000 tideland acres. Ninety percent of the reservation is bounded by water. There are approximately 900 enrolled Tribal members. The Swinomish, like their Indigenous Coast Salish relatives, are fishing, hunting and gathering people. With more than 26 species of fish and shellfish commonly found in their traditional fishing grounds, countless generations have been and continue to be integrally connected to the abundance of natural resources. Although most often associated with iconic salmon (all six species of Pacific salmon have runs in Swinomish fishing grounds), the Swinomish harvest a wide selection of flora and fauna from the land, sea and rivers. 

The project began following a Swinomish Senate meeting of elected leaders in which the first author discussed the results of a human health risk assessment of consuming local seafood. After presenting results summarizing individual physiological outcomes of elevated cancer and non-cancer risks at some of the sample sites due to contamination of bioaccumulative toxics, there was a delayed silence before the Chairman replied, “But what does this mean for the health of our community?” What followed was a discussion of the meaning of health and how conventional human health risk assessment methods had missed the mark by focusing only on individual, physiological health risks [10].

## 2. Materials and Methods 

### 2.1. Development of the IHI

Beginning in 2004, authors Larry Campbell (Swinomish staff and tribal elder) and Jamie Donatuto (Swinomish staff) collaborated with Swinomish community members, staff and leaders, and researchers outside the community (e.g., Robin Gregory, Terre Satterfield, Barbara Harper) to develop a first set of IHI. The authors organized interviews with over 100 Swinomish community members, with a focus on traditional, cultural resource users and elders. The authors conducted individual interviews with using an open-ended question format. Questions ranged from “what does ‘health’ mean to you?” to “describe impacts to ceremonies and gatherings if important natural resources are no longer available”. The questions gathered information on nonphysical health priorities, on community (as opposed to individual) health considerations, and on the potential impacts to these priorities if natural resources are damaged or destroyed. Author Donatuto coded the interview data, then cross-referenced the data with ethnographic records, published definitions of Indigenous health globally, and previous efforts to develop health measures for Indigenous peoples. The authors developed an evaluation that reflected the positive health values toward which a community strives, rather than negative indicators based on symptoms of ill health such as disease. Four initial Indigenous health indicators emerged: community cohesion, food security, ceremonial use, and education. The authors published a detailed account of the purpose, theories, methods and results in 2011 [10].

In 2009–2013, Tribal representatives from five other Coast Salish Tribes subsequently worked with the authors to expand the initial set of the IHI, providing a broader, Coast Salish representation of values and priorities. This regional Coast Salish group amended some of the indicator names and suggested two new indicators—for a total of six IHIs: community connection, natural resources security, cultural use, education, self-determination and resilience. In order to evaluate the new health indicators, the authors developed attributes [45] specific to Swinomish for each indicator. These attributes are useful in describing and filling out the meaning of each indicator (see Table 1). 

As an example, one of the Indigenous health indicators is “natural resources security.” Although numerous Swinomish members emphasized that this is an appropriate component of community health and well-being, it was not clear what the term signified and, as a result, it would not have been possible at a detailed level to identify how the community was likely to be impacted by certain types of changes to natural resources. In addition, the lack of detailed definition potentially opened the door to a serious error, which is that this indicator would be defined and then operationalized incorrectly by someone outside the community (e.g., a government agency or consultant) in a way that failed to match up with the values and preferences of the community members. In order to ensure that clear communication was established between the speakers and the assessment framework, the indicator therefore was described in greater detail by identifying three primary attributes: the *quality* of the resource; *access* to the resource by harvesters, and the extent to which harvesting, consuming and/or using the resource is perceived as “*safe*” by the community (e.g., absence of pollution). 

Once the authors summarized the initial set of IHI at these two levels—as indicators, and in terms of specific attributes—the next steps were to more closely define each of the IHIs using constructed measures and scales, and then to test the extent to which the measures were understandable to community members and accurately conveyed their health concerns. 

### 2.2. Developing Appropriate Scales

Descriptive scales provide measures that can potentially “fit” the demands of both western science and Indigenous cultures by creating a context-specific measure that reflects a health-based concern, often in the form of descriptive narratives or visuals linked to a numerical summary index (e.g., a scale of 1 to 4). Descriptive scales are useful when the subject matter defies simple quantification [40,45]. Researchers have created and used measures and scales in partnership with a number of Indigenous communities to demonstrate damages to cultural sites in Metis’ communities in Canada [46], for equitably revising flow regimes from hydroelectric dams [47], and to develop adaptive management strategies to reduce uncertainty in resource planning [40]. 

Some participants may resist ranking or assigning numbers to cultural or spiritual values associated with Indigenous health; descriptive scales allow for participants’ concerns to be prioritized and addressed in their own terms by combining a measure with narratives and other descriptive information (e.g., visual images, elders’ testimonies) [20]. The scales also allow for concerns and priorities to be clearly demonstrated without releasing proprietary knowledge that may be driving the chosen ranking on the scale. Descriptive scales offer a method that is easy to understand and employ; they are widely utilized for assessing a broad variety of objectives including the Gross National Product, the Dow Jones, and the APGAR scale for assessing newborn health. Ranking and weighting the indicators demonstrates the relative status of each indicator, and provides a baseline from which to compare future rankings of the same indicator (useful for illustrating health trend data over time). In addition, the rankings may allow for the establishment of thresholds indicating when negative impacts would occur, and for determining which variables predominantly influence the current health status [48].

### 2.3. Prioritizing Health Concerns

Once health concerns are identified, it is helpful to clarify their relative importance for the decision context at hand. Weighting contributions to health is difficult, particularly in Indigenous communities, because in a sense everything matters and is interconnected. For this reason, stating that one health attribute is more important than another can be perceived as neglecting some essential concerns. Yet not weighting concerns essentially assumes an equal weight among all health indicators, which may not accurately reflect the views of community members and can result in less robust health evaluations. 

There are many techniques for weighting across objectives or attributes; these include swing weighting and pair-wise comparisons [49]. Swing weighting has some conceptual advantages, in that it asks participants to imagine an attribute at its most healthy, then ‘weight’ the importance of how far away the attribute is from its ideal status in comparison to the other attributes. For example, even if a participant ranks each IHI as “looking good”, one or more of the IHIs may be higher priorities. Therefore, ranking *and* weighting provides a more complete picture of the status of each indicator as well as its relative importance in comparison with the other indicators. Weighting results are key for making informed decisions with limited time and resources because identify which indicators are the high priorities from the community’s point of view, irrespective of which indicators are in the poorest health. 

Pair-wise comparisons may be easier for participants to understand and complete. In pair-wise comparisons, two IHI are shown together and participants are asked to choose the more immediate priority on which to focus time and energy. The question is repeated until all IHI have been paired together (for 6 IHI, this is a total of 15 pairs). Both methods ask participants to think deeply about the value tradeoffs under consideration and thus have the potential to help lead to more thoughtful outcomes.

### 2.4. Study Design and Participants

In summer 2012, Swinomish hosted a workshop facilitated by the authors in order to test the IHI. The primary workshop goals were to find out if the IHI made sense to people, to see if they represented accurate depictions of Swinomish health, to determine whether the assessment methods were effective, and to test whether the ranking and weighting procedures were understandable and operational. The study design was reviewed and approved by the Tribe’s appointed Institutional Review Board (PAIHS #354449-1) prior to the workshop.

To ensure the participation and comfort of community members, authors Campbell and Donatuto led the recruitment by going door-to-door, phoning and emailing potential attendees. The authors sought willing participants with a wide variety of opinions, and thus did not attempt to secure a group representative of the Tribe’s demographics. Participants were sought from varying professions and ways of life in the Swinomish community.

**Figure 1:**
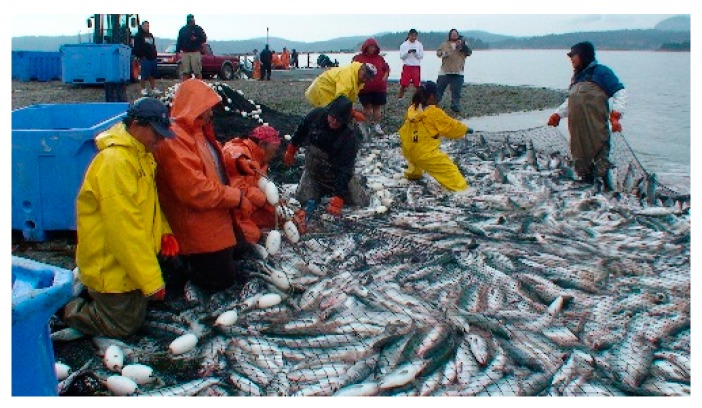
Image of Swinomish “community connection”—beach seiners pulling in a net with salmon. (photo by Jim Gibson).

Workshop discussions took place during a three-hour period, including a lunch of traditional foods. Questions were projected in PowerPoint, with TurningPoint^®^ polling software used to collect and display results. The polling software allowed answers to be tallied and recorded immediately via wireless, hand-held polling devices; simple statistics collated and visually depicted answers in the PowerPoint presentation (e.g., bar graph) while allowing individual responses to remain anonymous in a room full of familiar faces. Throughout the workshop, each of the six indicators was paired with a photograph depicting a common sight at Swinomish in order to provide a visual aid in describing the indicator and as a reminder of the indicator’s meaning. As one example, Figure 1 shows the photograph for “community connection;” the image depicts community members working together to haul in a net while beach seining salmon. 

Four different types of questions were asked of each participant. The first set of questions was demographic: age, gender, whether one lived on the reservation. The second set of questions, called “Where are we now?” established a baseline snapshot of the current community health in relation to the six indicators by answering a series of ranking questions using the IHIs. Participants ranked each indicator and attribute on a four-point descriptive scale (sample runs using a three- or five-point scale showed that the middle option was chosen most often; participants commented afterward that they felt health may actually lean more in one direction, but that it was easier to choose the “middle road” rather than give it more thought [50]. Therefore, the researchers adapted a four-point scale to compel participants to think through the question more carefully) developed in concert with Swinomish members:

1. Things are very bad
2. Not very good
3. Looking pretty good
4. We’re doing great

The third set of questions focused on ranking the attributes used to describe each indicator. For example, for “natural resources security”, participants ranked in order of most important to least important the three attributes of “access”, “quality”, and “safety”. In a fourth and final set of questions, workshop facilitators presented the group with two hypothetical yet realistic scenarios of local pollution events and asked to first rank and then weight (using swing weighting) which indicators would be most important to address first in light of the contamination. A first scenario described an on-reservation beach cleanup due to an oil spill; the second scenario described contamination in a widely fished river within the Tribe’s harvest area. These two scenarios are shown below.

Scenario 1: Beach cleanup
  A cleanup is underway at a beach located on the reservation. For many years, this beach has been a popular steamer clam digging area for tribal members, who also value it for ceremonial and spiritual purposes. A recent oil spill from a passing tanker has contaminated the beach, forcing tribal members to travel to a remote beach in order to dig. The clams at the remote beach are mostly cockles (not a preferred species by Swinomish people). Despite media attention, no cleanup has occurred. The tanker owners—the business responsible—are bankrupt. State and Federal authorities have promised a full cleanup, but they lack funds and there is a backlog of other cleanup sites, making it unlikely that the spill will be cleaned up quickly or 100%. The State and Federal governments are now asking for help from Tribal members to provide guidance on where to focus the limited resources and funding. The Tribe has agreed to provide information about the effects of the beach spill on six community health indicators.
Scenario 2: River cleanup
  The Tribe’s main fishing river is being polluted by intensive farming practices, including agricultural run-off, livestock entering the river, and deforestation of the riverbanks. Some community members are worried that pollution in the river is negatively affecting the health of Elders and children in the community, and negatively impacting the numbers and health of juvenile salmon, affecting not only the food fishery but also the use of salmon for ceremonial purposes. The Tribe plans to exercise its legal rights by pushing for better protection of the river, but changing regulations and behaviors to limit pollution and protect the river’s banks will take a long time. In the short term, local and State regulators insist that, although they support the Tribe’s concerns, they do not have enough money or staff to begin to clean up the river. The Tribe is frustrated and is seeking input from community members to prioritize restoration efforts with the goals of reducing health risks to the human community and maintaining and protecting salmon habitat.

## 3. Results

In July 2012, sixteen adult tribal members participated in the workshop. Seventy percent of the participants were female and the large majority of the participants lived on Reservation. Table 2 displays the ages of the participants. 

**Figure 2:**
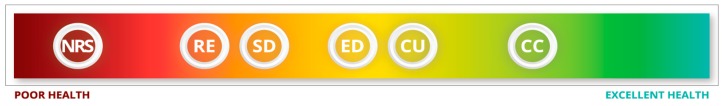
Workshop participants’ views on the status of community health using the Indigenous Health Indicators. (NRS = natural resources security; RE = resilience; SD = self-determination; ED = education; CU = cultural use; CC = community connection).

The workshop data and participants’ comments demonstrate that the IHIs help depict key non-physiological health priorities and concerns. An overview of the results are presented here to illustrate the usefulness of the methods; detailed data are not given because the results are not representative of the Tribe and the authors do not want to misrepresent the current health status of the community. For the “Where are we now?” health status questions, the collated results depicted respondents’ views that each of the IHIs are in a unique position in on the continuum of poor health to excellent health. None of the IHIs were uniformly ranked as “very bad” or “great”, and none of the IHIs were ranked as having the same status. The distinctions between the status of each of the indicators, as well as differences in how they were ranked by the workshop participants, points to the efficacy of the IHIs representing unique aspects of health that are of importance to participants. Figure 2 illustrates a summary of the “where are we now?” health status results. To determine the summary statistics, the ranking results for all measures were added for one of the IHI, then the IHIs were compared. For example, for natural resources security, the descriptive rankings (i.e., “we are doing great, looking pretty good, not very good, and things are very bad”) were summed for the three measures: quality, access, and safety. The majority of respondents chose “not very good” as the status of all three measures (access, abundance, sharing) in differing percentages. The total number of “not very good” rankings, versus the total number of the other three rankings, reflects a general snapshot of workshop participants’ beliefs regarding the health status of the natural resources security IHI, thus putting natural resources security in the “not very good” category overall. While this method obscures the measures of the individual measures for natural resource security, which are important in an actual evaluation, the individual measure results are not integral to test whether the method works. Figure 2 depicts natural resources security as the IHI with the lowest health status on the health continuum.

None of the indicators was unanimously ranked as the least important, which shows that all of the indicators resonate with at least one of the workshop participants. Had an indicator been uniformly considered “least important”, researchers would need to rethink that indicator—its meaning, purpose and inclusion in the IHI set. In an actual evaluation, the ranking exercise provides insights to the health priorities, irrespective of the health status of that indicator, which is important information for decision-makers. For example, if community members had ranked restoration programs (a Self-Determination attribute) as in very poor health and the least important, while healing programs (a different Self-Determination attribute) in very poor health but the most important, then decision-makers with limited resources can choose to focus on healing programs rather than restoration.

**Figure 3:**
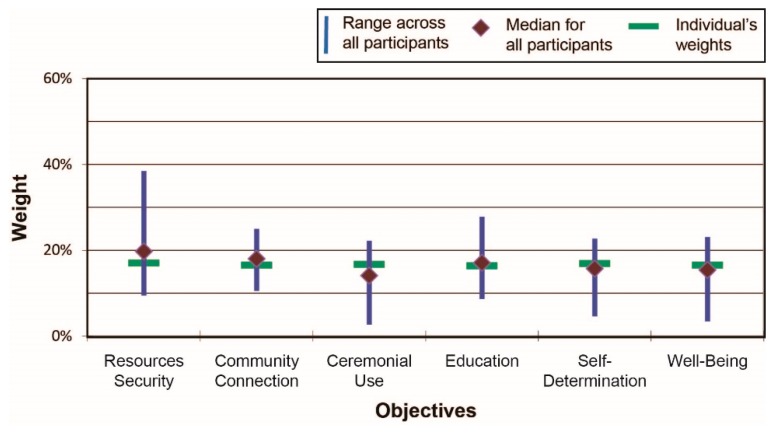
Scenario 1 (beach clean-up) results (At the time of the workshops, the “Resilience” indicator was called “Well-Being”.).

**Figure 4:**
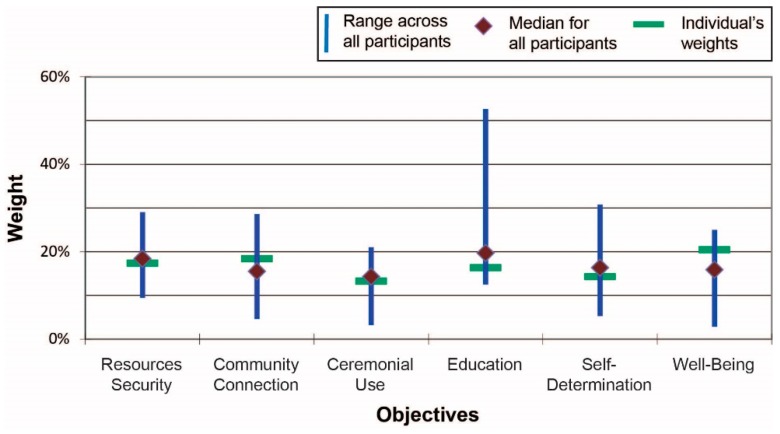
Scenario 2 (river clean up) results (At the time of the workshops, the “Resilience” indicator was called “Well-Being”.).

The fourth and final part of the workshop—ranking and weighting the scenarios—proved to be the most arduous. Several participants verbally commented that it was difficult for them to understand the swing weighting concept. Other participants noted that ranking and weighting indicators are cognitively and emotionally challenging tasks, yet understood that if ranking and weighting are not done, then by default all 18 measures across the six different indicators are assumed to have equal importance. Figure 3 shows results across all participants for Scenario 1 (beach cleanup), including the scenario-specific range of weights across all participants for the six IHI indicators along with the median values (depicted by the diamond shapes) and the weights assigned by one (anonymous) individual. Several indicators, such as natural resources security, varied substantially across participants in terms of assigned importance; other indicators, such as community connections, were assessed more similarly by participants. Figure 4 presents the summary results for Scenario 2 (river cleanup). As is clearly shown, the fourth indicator—education—varied considerably in how it was valued by participants relative to the other indicators: on average it was rated slightly higher than any other indicator but the range of responses is high. There was substantial agreement, on the other hand, with respect to the relative importance placed on ceremonial uses of resources and concerns related to resources security.

At the end of the workshop, facilitators held an open discussion with participants about the purpose and goals of the workshop, methods used, clarity of the questions and relevance for community concerns and priorities. Overall, participants were supportive of the use of the IHIs and understood that the results could be helpful in understanding community health needs. There was general agreement among participants that not all the indicators and attributes were equally important; instead, it was clear from the scenarios exercise that the type and context of a proposed initiative (what type of actions might take place, what types of resources might be affected, what parties would be in charge) made a difference. 

Three criteria were established for gauging the success of the IHI workshop approach:

- Do the rankings/weightings make sense in terms of expressing accurately what participants feel is important?
  Based on participants’ willingness to complete the questions and the positive feedback received after the workshop, the answer appears to be yes.
- Are there distinctions among participants in terms of how the indicators were ranked?
  Looking over the between-subject data (not shown here), the answer is clearly yes.
- Are there distinctions across the two test cases?
  With reference to the relative weights for each case, the answer is yes.

Because these results are illustrative and serve only as a test of IHI evaluation methods, we underscore that these data should not be used as evidence of generalized relationships among the indicators. However, based on the results, it is clear that this exploratory set of health indicators was able to distinguish both between scenarios and among the six different measures of value. An identical result for the two scenarios would not demonstrate sufficient sensitivity in the measures. Similarly, an assumption of equality among the IHIs—the default option if explicit weightings were not conducted—would not present an accurate picture of the views of community members. 

## 4. Discussion

Although the concept of defining and evaluating health based on the beliefs and values of the people in question is not new, the majority of Indigenous-related health assessments have done relatively little in terms of developing measures of the cultural and social aspects of community conceptions of health [8,9,10,12,13,14,15,16,17,18]. There are a handful of initiatives that demonstrate how appropriate Indigenous health assessments can be done, e.g., the Maori HIA [27] and the Canadian longitudinal health survey [29]. However there are no widely accepted methods for Indigenous community health assessments that are simple to use, understand and tailor for individual, unique communities and contexts. This is where the IHI may be able to play a role. 

Incorporating Indigenous knowledge with other knowledge systems has been problematic historically [32,33]. Therefore, caution is advised with respect to the proposed framework. The authors acknowledge that IHI findings cannot relay the nuance or complexity needed to depict the full range of deep-seated values, beliefs and relationships that they represent. Taken out of context, Indigenous knowledge can be misrepresented, misunderstood, or both. By making use of constructed scales to describe some of the key nonphysical, community-based indicators of Indigenous health, the goal is to provide an equal playing field, from both western and Indigenous perspectives, about past, present and future changes. 

Indigenous communities have a right to have their knowledge and their perspective inform any project assessment that could affect their health, environmental, economic, social, or cultural well-being. The United Nation’s State of the World’s Indigenous Peoples Report identifies four elements that should characterize Indigenous health indicators: (1) basing the indicators on Indigenous meanings of health; (2) acknowledging the community’s right to self-determination; (3) full and effective participation by community members in decision-making arenas (based on the principle of free, prior and informed consent); and, (4) the recognition and protection of collective Indigenous knowledge [51]. The development of the IHI has attempted to reflect these four concerns and thereby aid Indigenous communities to gather and assess information needed to identify their own health, well-being and development goals. 

In the case of U.S. federally recognized Tribes, the integration of Indigenous health concerns and priorities is consistent with the legal responsibility of the U.S. federal government. Tribes hold treaty-secured rights that ensure health for themselves and their natural resources. The U.S. federal government’s trusteeship includes responsibilities to assess and define health as the Tribes do [52]. Although the federal government has acknowledged this responsibility (cf. U.S. Executive Order 13175), most agencies have yet to effectively enact changes in health-related regulations such as the human health risk assessment and management paradigm [14]. It is the hope that the ideas and the IHI set contained in this paper will help to advance this long-overdue process. The authors have actively disseminated project results via multiple presentations at conferences and on webinars, and on the Swinomish website (www.swinomish.org/IHI/).

Since the initial indicator pilot workshop with Swinomish, other Indigenous communities have participated in trials and uses of the IHIs [53,54,55]. Results show that the methods are effective with disparate groups (disparate in the sense that each Tribe is unique and that even neighboring communities have different health priorities). In each case, results have been presented to respective Tribal Councils for review and approval before being released (based on material data sharing plans [56]). Verbal presentations provide each Council with a summary of the results as well as some potential uses for the IHIs: establishing baseline health status, emergency preparedness plans, setting cleanup guidelines, and a host of other health-related policies, both on and off Reservation, with the caveat that the indicator set is still in the testing phase. The results and feedback to date from communities have been positive and supportive, with ongoing discussions about how the IHIs could be furthered refined and incorporated into decision-making practices and policies. 

## 5. Conclusions

To date, Indigenous community health assessment methods have not included many of the “intangible” aspects of Indigenous community health (Objective a). This paper describes a community-based approach using multiple methods (individual interviews, group workshops, ranking with descriptive scales, weighting techniques) for identifying more detailed, context-specific Indigenous health risks and impacts (Objective b). A collaborative group of some Coast Salish tribal communities developed an innovative set of Indigenous Health Indicators (IHIs) for evaluating Indigenous priorities (Objective c). Results from pilot-tests of the IHI with the Swinomish Indian Tribal Community (Washington State, USA) show that the IHI can effectively elucidate some of the “intangible” aspects Indigenous community health (Objective d). 

The six IHIs (community connection, natural resources security, cultural use, education, self-determination, and resilience) advanced in this paper reflect multiple connections between Indigenous community members and between community members and the non-human world. As an example, the “cultural use” indicator recognizes related activities as integral contributors to culture, health, governance, and spirituality. For Swinomish, as with many Indigenous communities, the core cultural practices rely on local natural resources, characterized by respectful, appropriate harvest and preparation that takes considerable time, thoughtful intention, and collaboration [57,58]. In many cases there will be strong linkages among the identified health indicators. However—keeping in mind both the practical intent of the approach and the trial nature of these results—this does not preclude adopting specific definitions for each of the six IHIs, as refined by each community, in order to establish the basis for a more complete and defensible health assessment. The intended outcome is a set of IHIs that contribute to development of an improved, more comprehensive Indigenous health assessment framework and provide specific attributes and measures that can be modified to fit the practices, beliefs and values reflective of each community’s health meanings and nuances, while retaining the broader IHI key indicator categories as a consistent framework with which to inform and amend policies and regulations.

## Figures and Tables

**Table 1:** Indigenous Health Indicators and respective attributes.

**Community Connection**
**Work**—Community members have a job or role that they and other community members respect and they work together (mutual appreciation, respect, cooperation).
**Sharing**—Community members engage in active sharing networks, which are integral to a healthy community, ensuring that everyone in the community receives traditional foods and other natural resources such as plant medicines, especially Elders.
**Relations**—Community members support, trust and depend on each other.
**Natual Resources Security**
**Quality**—The natural resources, including the elements (e.g., water), are abundant and healthy.
**Access**—All resource use areas (i.e., Usual and Accustomed areas in WA) are open to harvest/use (not closed or privatized) by community members.
**Safety**—The natural resources themselves are healthy, not affected by pollution, climate change, etc.
**Cultural Use**
**Respect/Stewardship**—Community members are conferring respect of/to the natural resources and connections between humans, environment and spirit world; ensuring cultural resources are properly maintained.
**Sense of Place**—Community members are engaging in traditional resource-based activities, which is a continued reminder/connection to ancestors and homeland.
**Practice**—Community assemblies able to follow appropriate customs (e.g., can obtain specific natural resources if needed such as cedar, certain foods, etc.), and are able to honor proper rituals, prayers and thoughtful intentions. Community members feel that they are able to satisfy spiritual/cultural needs, e.g., consume foods and medicines in order to satisfy Spirit’s “hunger”.
**Education**
**The Teachings**—The community maintains the knowledge, values and beliefs important to them.
**Elders**—The knowledge keepers are valued and respected, and able to pass on the knowledge.
**Youth**—The community’s future is able to receive, respect, and practice the Teachings.
**Self-Determination**
**Healing/restoration**—The availability of and access to healing opportunities (e.g., traditional medicines, language programs) for community members, as well as the community’s freedom to define and enact their own, chosen environmental, health, and habitat restoration programs.
**Development**—The ability for a community to determine and enact their own, chosen community enrichment activities in their homelands without detriment from externally imposed loss of resources.
**Trust**—The community trusts and supports its government.
**Resilience**
**Self-Esteem**—The beliefs and evaluations community members hold about themselves are positive, providing an internal guiding mechanism to steer and nurture people through challenges, and improving control over outcomes.
**Identity**—Community members are able to strongly connect with who they are as a community (Tribe or Nation) in positive ways.
**Sustainability**—The community is to adapt (e.g., people hunt with guns and use motorboats today but that doesn’t discount the significance of harvesting) and move within homelands voluntarily in response to changes (the “7 generations thinking”).

**Table 2:** Age of workshop participants.

Under 20	1
21–30	1
31–40	2
41–50	2
51–60	3
61–70	5
71+	2
Total	16

## Abbreviations

The following abbreviations are used in this manuscript:

IHI	Indigenous Health Indicators
HIA	health impact assessment
HHRA	human health risk assessment
CHA	community health assessment
